# Clinicopathological and CT features of tumor spread through air space in invasive lung adenocarcinoma

**DOI:** 10.3389/fonc.2022.959113

**Published:** 2022-09-23

**Authors:** Lili Qin, Yubing Sun, Ruiping Zhu, Bo Hu, Jianlin Wu

**Affiliations:** ^1^ Graduate School of Tianjin Medical University, Tianjin, China; ^2^ Department of Radiology, Dalian Public Health Clinical Center, Dalilan, China; ^3^ Department of Radiology, Affiliated Zhongshan Hospital of Dalian University, Dalilan, China; ^4^ Department of Interventional, Affiliated Xinhua Hospital of Dalian University, Dalilan, China; ^5^ Department of Pathology, Affiliated Zhongshan Hospital of Dalian University, Dalilan, China; ^6^ Department of Pathology, Affiliated Xinhua Hospital of Dalian University, Dalilan, China

**Keywords:** lung cancer, adenocarcinoma, radiology, spread through air space, computed tomography

## Abstract

**Objective:**

Tumor spread through air spaces (STAS) has recently been reported as a novel invasive pattern in lung adenocarcinoma. Thus, this study aimed to investigate the clinicopathological and radiological features in invasive lung adenocarcinoma with tumor STAS.

**Methods:**

Data of 503 invasive lung adenocarcinoma patients who underwent surgery between 1 January 2015 and 31 December 2021 were collected. The correlations between STAS presence and clinicopathological and radiological characteristics were analyzed. Statistical analysis was performed using SPSS 22.0.

**Results:**

Among the 503 patients with invasive adenocarcinoma, 247 (47.9%) and 262 (52.1%) patients were positive and negative for STAS, respectively. Compared to STAS-negative adenocarcinoma, STAS was more common in papillary, micropapillary, and solid tumors (p < 0.01); STAS was associated with advanced pT (p = 0.024), pN (p < 0.001), and pTNM (p < 0.001) stage, more lymph node metastases (p < 0.01), more pleural invasion (p < 0.01), and more neurovascular invasion (p = 0.025). The maximum diameter (p < 0.01), the maximum diameters of the solid component (p < 0.01), and the consolidation/tumor ratio (CTR, p < 0.01) were significantly larger in STAS-positive than in STAS-negative adenocarcinoma. Other common computed tomography (CT) features of adenocarcinomas, i.e., lobulation (p < 0.01), spiculation (p < 0.01), vacuole (p < 0.01), air bronchogram (p = 0.020), vascular convergence (p < 0.01), and pleural indentation (p < 0.01) were significantly associated with STAS. In a multivariable analysis, the maximal diameter of the solid component (odds ratio [OR], 2.505; 95% confidence interval [CI], 1.886–3.329), vacuole (OR, 3.301; 95% CI, 1.822–5.980), and spiculation (OR, 2.162; 95% CI, 1.221–3.829) were independent predictors of STAS. The area under the curve (AUC) of the maximal diameter of the solid component was 0.757 (95% CI, 0.714–0.799; p < 0.001), the sensitivity was 73.9%, and the specificity was 69.1% at a cutoff value of 1.18 cm.

**Conclusion:**

STAS was significantly correlated with several invasive clinicopathological and radiological characteristics, and the maximal diameter was an independent predictor of STAS. These results will prove helpful in identifying STAS-positive adenocarcinoma by CT before surgical resection.

## Introduction

In the 2015 World Health Organization (WHO) classification of lung adenocarcinoma (1), tumor spread through air spaces (STAS) was recognized as an invasive pattern of lung adenocarcinoma, which was defined as micropapillary clusters, solid tumor nests, or single tumor cells spread beyond the edge of the tumor into air space surrounding the lung parenchyma. This was a further advancement in the research and understanding of the histopathological characteristics of lung adenocarcinoma, having important clinical significance. Several studies have shown that STAS is significantly associated with the postoperative prognosis of lung cancer and is a major risk factor for early-stage lung adenocarcinoma after local resection (2–4). However, STAS can only be diagnosed by post-surgical pathological observation, making it difficult to provide guidance on preoperative treatment plans. Therefore, it is a challenge to preoperatively assess and predict STAS using information from imaging data. This study retrospectively collected 503 cases of invasive lung adenocarcinoma and systematically analyzed the clinical, pathological, and computed tomography (CT) features associated with STAS to explore imaging markers that could help predict STAS.

## Materials and methods

The Research Ethics Committee of Zhongshan Hospital Affiliated to Dalian University (Dalian, China) approved this study (project approval number 2021029) and waived informed consent for this retrospective study.

### Patients

We retrospectively examined patients who underwent surgical resection of primary lung adenocarcinoma between January 2015 and December 2020. Medical records and archival slides were analyzed. Cases with other malignant tumors and neoadjuvant therapy, other lung cancer surgery within the past 2 years, positive surgical margin, and no available tumor slides or CT images for review were excluded from the study cohort. According to these criteria, we identified a total of 503 patients. The clinical information included age, sex, smoking history, blood type, and carcinoembryonic antigen (CEA) levels.

### Histologic evaluation

All surgically resected specimens were fixed with 10% formalin, embedded in paraffin, sliced to a thickness of 5 mm, and stained with hematoxylin and eosin (HE). Tumor slides were reviewed by two specialists with more than 10 years of experience in pathological diagnosis, of whom both were blinded to patient clinical outcomes. At least three slides of each case were reviewed. In case of disagreement, a consensus was reached after discussion.

**Figure 1:**
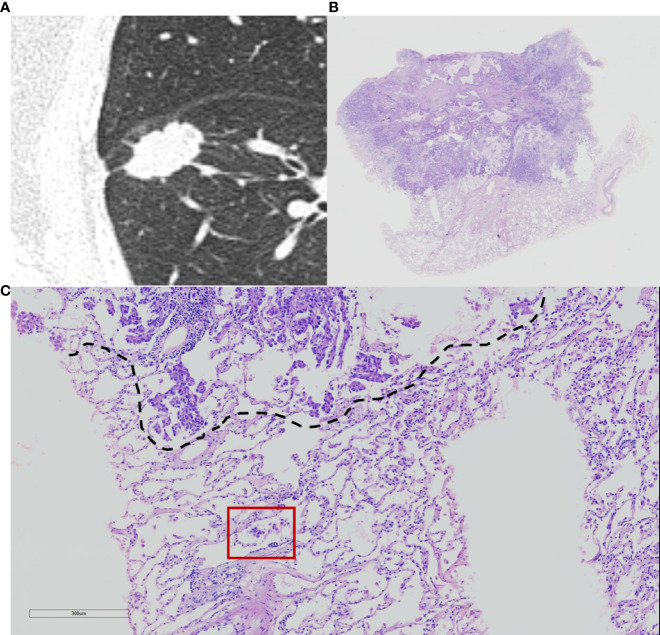
Computed tomography (CT) and histology images of spread through air space (STAS)-positive lepidic lung adenocarcinoma. **(A)** CT image (width, 1,500 HU; level, −600 HU) shows a solid nodule with lobulation, spiculation, and pleural indentation signs; **(B)** photomicrograph shows a distinct border between the solid tumor and lung parenchyma (hematoxylin and eosin [HE] stain, ×10); **(C)** micropapillary pattern of STAS (red square) identified within air spaces in the lung parenchyma beyond the edge (dashed line) of the main tumor (HE stain, 200×).

**Figure 2:**
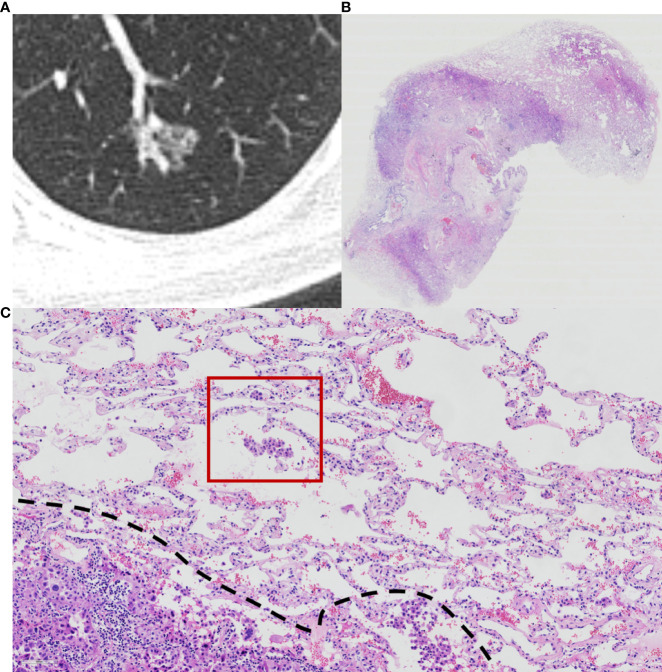
Computed tomography (CT) and histology images of spread through air space (STAS)-positive acinar-pattern lung adenocarcinoma. **(A)** Axial CT image (width, 1,500 HU; level, −600 HU) shows a partly solid nodule with lobulation and vascular convergence; **(B)** photomicrograph shows a distinct border between the solid tumor and lung parenchyma (hematoxylin and eosin [HE] stain, ×10); **(C)** photomicrograph shows detached micropapillary clusters and solid nests of tumor cells in alveolar tissue beyond the edge (dashed line) of the main tumor (HE stain, 200×).

Histopathological subtype (WHO Classification 2015) (1), pathologic tumor node metastasis (TNM) stage (8th edition of the Lung Cancer Staging System) (5), lymph node metastasis, pleural invasion, neurovascular invasion, and STAS status data were collected. STAS was defined as the tumor cells observed in the air space in the surrounding lung parenchyma beyond the edge of the main tumor without any direct connection between the detached tumor cells and the main tumor. The edge of the main tumor was defined as the surface of the tumor that is easily recognizable at low-power field examination as highlighted with the dotted line in **Figures 1** and **2**. Tumor cells outside this line were considered as STAS when no contact to the main tumor mass.

### CT acquisition and interpretation

All patients underwent chest CT within 2 weeks before surgery. Examinations were performed by one of these three scanners: Siemens Somatom Definition AS 128, Siemens Somatom Definition AS 64, and Siemens Somatom Definition. Patients were scanned in the supine position with arms raised and head advanced after inspiration and breath-hold from the thoracic inlet to the posterior rib diaphragm angle. Scanning parameters were tube voltage of 80–120 kV, automatic tube current, slice thickness of 5 mm, and matrix of 512 × 512. Image data were reconstructed with 1-mm-thick sections with the lung algorithm. Two radiologists with more than 10 years of experience in chest image interpretation analyzed CT images, and the consensus was reached by discussion in case of disagreement. Both radiologists were blinded to the STAS status of patients.

At 1,500 HU window width and −600 HU window level on the lung reconstructions, CT image features were analyzed, including tumor location, tumor density (solid, subsolid, and ground glass), the maximal diameter of the tumor, the maximum of the solid component, consolidation/tumor ratio (CTR), lobulation, spiculation, cavitation, air bronchogram, pleural indentation, and vascular convergence.

### Statistical analyses

SPSS 23.0 software (SPSS Statistics, IBM, Chicago, IL, USA) was used for statistical analysis. All cases were divided into STAS-positive and STAS-negative groups. Clinical, pathological, and CT features between the two groups were compared by using the χ**^2^** test or Fisher exact test for categorical variables and t-test or Mann–Whitney U test for continuous variables. The variables with a p-value of <0.1 in univariate analysis were included in logistic regression for multivariate analysis, and a p-value of < 0.05 was considered statistically significant. Multivariate model was developed by using multifactorial logistic regression based on clinical and CT features.

## Results

### Association between STAS and clinical features

A total of 503 patients with pathologically confirmed invasive lung adenocarcinoma, with an age range of 28–82 years and a mean age of 61.16 ± 8.99 years, were recruited for this study. There were 241 cases (47.9%) in the STAS-positive group, of which 98 (40.7%) were male, and 143 (59.3%) were female, with a mean age of 62.19 ± 8.831 years. Of these, 45 (18.8%) patients had a history of smoking. There were 262 cases (52.1%) in the STAS-negative group, of which 103 (39.3%) were male, and 159 (60.7%) were female, with a mean age of 60.21 ± 9.041 years. Of these, 43 (16.4%) patients had a history of smoking. The difference in age between patients in the STAS-positive and STAS-negative groups was statistically significant (p = 0.015), while the differences in gender, blood group, CEA, and smoking history were not statistically significant (p = 0.757, 0.208, 0.075, and 0.491, respectively). The clinical characteristics of our study are summarized in **Table 1**.

**Table 1:** Clinical features of 503 patients with invasive lung adenocarcinoma (n [%]).

Variable	All patients N = 503	STAS positive N = 241 (%)	STAS negative N = 262 (%)	t/x²	p*-*value
Age (y)	61.16 ± 8.99	62.19 ± 8.831	60.21 ± 9.041	2.472	0.015
Sex				0.095	0.757
Male	201 (40.0)	98 (40.7)	103 (39.3)		
Female	302 (60.0)	143 (59.3)	159 (60.7)		
Smoking status				0.444	0.505
Former or current	88(17.5)	45 (18.8)	43 (16.4)		
Never	415(82.5)	196 (81.3)	219 (83.6)		
Blood type				4.54	0.208
A	135 (26.8)	67 (27.8)	68 (25.9)		
B	153 (30.4)	71 (29.3)	82 (31.3)		
O	156 (31.0)	68 (28.2)	88 (33.6)		
AB	59 (11.7)	35 (14.5)	24 (9.2)		
CEA (ng/mL)	4.267 ± 16.33	6.03 ± 22.85	2.51 ± 2.00	1.796	0.075

STAS, spread through air space; CEA, carcinoembryonic antigen.

### Association between STAS and histological characteristics

As shown in **Table 2**, the presence or absence of STAS was significantly associated with the histologic subtype of invasive lung adenocarcinoma, and the proportion of papillary, micropapillary, and solid subtypes was significantly higher in the STAS-positive group than in the STAS-negative group (19.5% vs. 4.2%; 2.5% vs. 0.4%; 3.7% vs. 0.8%, respectively, p < 0.01). Especially when there were micropapillary structures in the tumor, the positive rate of STAS was significantly higher (p = 0.002). Additionally, STAS was also significantly correlated with TNM staging of lung adenocarcinoma. In lung adenocarcinoma of T2, N2, and IB stages, the positive rate of STAS was higher, and the difference was statistically significant (p = 0.024, p < 0.001, p < 0.001). In lymph node metastasis, pleural invasion, and neurovascular invasion, there were statistically significant differences between the STAS-positive and STAS-negative groups (all p < 0.05).

**Table 2:** Association between STAS and pathological characteristics (n [%]).

Variable	All patients N = 503	STAS positive N = 241 (%)	STAS negative N = 262 (%)	t/x²	p*-*value
T stage				6,635	0.024
T1	454 (90.3)	209 (86.7)	245 (93.5)		
T2	46 (9.1)	30 (12.4)	16 (6.1)		
T3	3 (0.6)	2 (0.8)	1 (0.4)		
N stage				15.530	<0.001
N0	463 (90.3)	211 (87.6)	252 (96.2)		
N1	21 (4.2)	14 (5.8)	7 (2.7)		
N2	19 (3.8)	16 (6.6)	3 (1.1)		
Pathological stage				13.113	<0.001
IA	426 (84.7)	186 (77.2)	240 (91.6)		
≥IB	77 (15.3)	55 (22.8)	22 (8.4)		
Histologic subtype				73.088	<0.001
Lepidic	135 (26.8)	30 (12.4)	105 (40.1)		
Acinar	272 (54.1)	136 (56.4)	136 (51.9)		
Papillary	58 (11.5)	47 (19.5)	11 (4.2)		
Micropapillary	7 (1.4)	6 (2.5)	1 (0.4)		
Solid	11 (2.2)	9 (3.7)	2 (0.8)		
Mucinous	20 (4)	13 (5.4)	7 (2.7)		
Micropapillary feature				9.430	0.002
Present	12 (2.4)	11 (4.6)	1 (0.4)		
Absent	491 (97.6)	230 (95.4)	261 (99.6)		
Lymph node metastasis				12.298	<0.001
Present	75 (14.9)	30 (12.4)	10 (3.8)		
Absent	463 (92)	211 (87.6)	252 (96.2)		
Pleural invasion				18.306	<0.001
Present	122 (24.3)	79 (32.8)	43 (16.4)		
Absent	381 (75.7)	162 (67.2)	219 (83.6)		
Neurovascular invasion				4.993	0.028
Present	26 (5.2)	18 (7.5)	8 (3.1)		
Absent	477 (94.8)	223 (92.5)	254 (96.9)		

STAS, spread through air space.

### Association between STAS and CT features

Among the 503 cases in this study, pure ground-glass nodules were found in 110 cases (21.9%), subsolid nodules were found in 193 cases (38.4%), and solid nodules were found in 200 cases (32.2%), of which solid nodules had a significantly higher rate of STAS positivity (p < 0.01). The maximal diameter of tumor, the maximal diameter of solid components, and CTR in the STAS-positive group were higher than those in the STAS-negative group, and the differences were statistically significant (all p < 0.01). Additionally, lobulation (p < 0.01), spiculation (p < 0.01), cavitation (p < 0.01), air bronchogram (p = 0.020), pleural indentation (p < 0.01), and vascular convergence (p = 0.001) were also more common in the STAS-positive group than in the STAS-negative group, and all differences were statistically significant. The CT features of the study are summarized in **Table 3**.

**Table 3:** Association between STAS and CT features (x̅±s/n [%]).

Variable	All patients N = 503	STAS positive N = 241 (%)	STAS negative N = 262 (%)	t/x²	p-value
Location				2.460	0.652
RUL	165 (32.8)	72 (29.9)	93 (35.5)		
RML	31 (6.2)	14 (5.8)	17 (6.5)		
RLL	97 (19.1)	51 (21.2)	46 (17.6)		
LUL	123 (24.5)	62 (25.7)	61 (23.3)		
LLL	87 (17.3)	42 (17.4)	45 (17.2)		
Tumor density				54.441	<0.01
ground glass	110 (23.1)	19 (7.9)a	91 (34.7)b		
subsolid	193 (38.4)	103 (42.7)a	123 (34.4)a		
solid	200 (32.2)	119 (49.4)a	81 (30.9)b		
Maximum tumor diameter (cm)	2.13 ± 0.43	2.47 ± 1.02	1.82 ± 0.81	7.876	<0.01
Maximum solid component diameter (cm)	1.37 ± 0.52	1.89 ± 1.12	0.90 ± 0.97	100.136	<0.01
CTR	0.61 ± 0.02	0.75 ± 0.315	0.47 ± 0.42	8.235	<0.01
Lobulation				40.287	<0.01
Present	312 (62.0)	184 (76.3)	128 (48.9)		
Absent	191 (38.0)	57 (23.7)	134 (51.1)		
Spiculation				51.113	<0.01
Present	175 (34.8)	122 (50.6)	53 (20.2)		
Absent	328 (65.2)	119 (49.4)	209 (79.8)		
Cavitation				20.256	<0.01
Present	109 (21.7)	73 (30.3)	36 (13.7)		
Absent	394 (78.3)	168 (69.7)	226 (86.3)		
Air bronchogram				5.370	0.020
Present	203 (40.4)	110 (45.6)	93 (35.5)		
Absent	300 (59.6)	131 (54.4)	169 (64.5)		
Pleural indentation				27.388	<0.01
Present	219 (43.5)	134 (55.6)	85 (32.4)		
Absent	284 (56.5)	107 (44.4)	177 (67.6)		
Vascular convergence				11.727	0.001
Present	414 (82.3)	213 (88.4)	201 (76.7)		
Absent	89 (17.7)	28 (11.6)	61 (23.3)		

RUL, right upper lobe; RML, right middle lobe; RLL, right lower lobe; LUL, left upper lobe; LLL, left lower lobe, CTR, consolidation/tumor radio.

### Multivariate regression analysis

Multivariate logistic regression analysis showed that the maximal diameter of solid components on CT, spiculation, and cavitation were independent predictors of STAS in invasive lung adenocarcinoma, as detailed in **Table 4**. However, other factors that were statistically different in the univariate analysis, such as tumor density, CTR, and fractional sign, were not statistically significant in the multivariate analysis.

**Table 4:** Multivariable logistic analysis results.

Variable	β	S.E.	Wald	p-value	OR	95%CI	
Maximum solid component diameter	0.918	0.145	40.124	0.000	2.505	1.886	3.329
Spiculation	0.771	0.292	6.988	0.008	2.162	1.221	3.829
Cavitation	1.194	0.303	15.508	0.000	3.301	1.822	5.980
Tumour density			9.172	0.010			
Subsolid	0.631	0.410	2.366	0.124	1.879	0.841	4.196
Solid	−0.23	0.511	0.202	0.653	0.795	0.292	2.163
Age				0.743			
CEA				0.219			
Maximum tumour diameter				0.698			
CTR				0.221			
Lobulation				0.300			
Air bronchogram				0.485			
Pleural indentation				0.942			
Vascular convergence				0.612			

OR, odds ratio.

The receiver operating characteristic curve (ROC) analysis of the above variables found that the area under the curve (AUC) of the maximal diameter of the solid component was 0.757 (95% CI, 0.714–0.799) when the cutoff value was 1.18 cm, the sensitivity was 73.9%, and the specificity was 69.1%.

**Figure 3:**
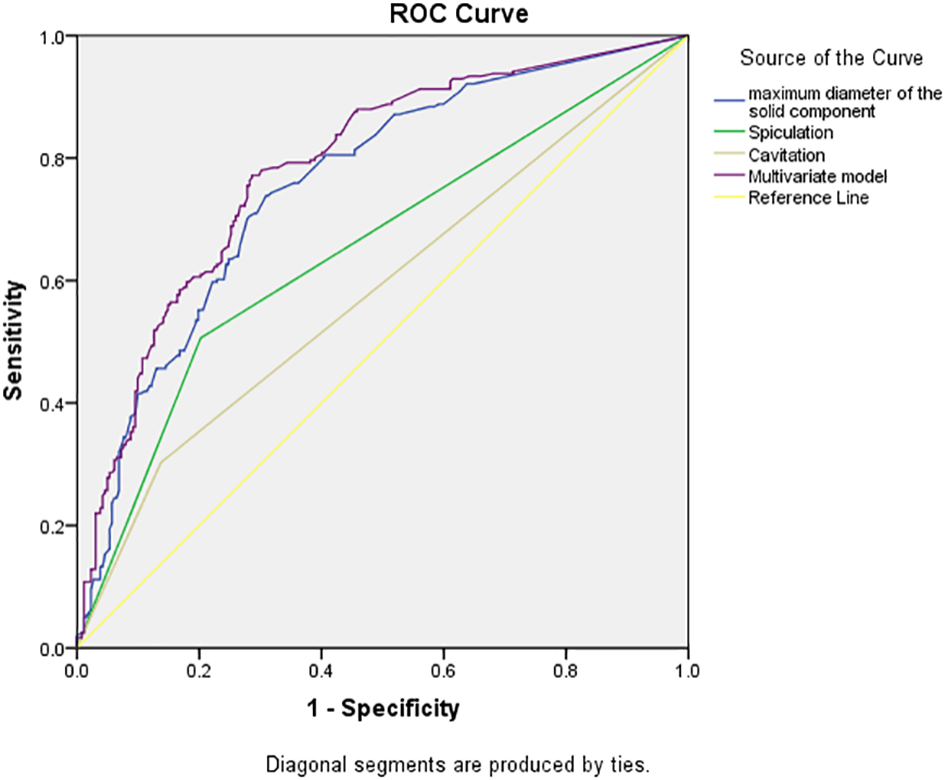
Receiver operating characteristic curves of independent predictors and multivariate model of spread through air space (STAS). The area under the curve of the multivariate model was 0.785 (95% confidence interval: 0.745–0.825, P < 0.001).

Multivariate model was established on maximum diameter, spiculation, and cavitation. As is shown in **Figure 3**, the performance of multivariate model is better than any single predictor. The AUC of multivariate model was 0.785 (95% CI, 0.745–0.825), when the cutoff value was 0.48, the sensitivity was 77.2%, and the specificity was 71.4%.

## Discussion

Primary lung cancer remains a common malignant tumor with high morbidity and mortality worldwide. Authoritative organizations, such as the WHO, revise the histopathological classification of lung tumors about every 5 years. In the 2015 WHO Classification of Lung Cancer, STAS was officially proposed as a new invasive mode of lung adenocarcinoma. This has undoubtedly been of great practical importance to the understanding of the biological behavior of lung adenocarcinoma and guiding the clinical treatment and assessment of prognosis, immediately attracting widespread attention from researchers (2, 6–9). Several studies (3, 4, 10–12) have shown that STAS is significantly associated with the prognosis of lung adenocarcinoma, i.e., overall survival and recurrence-free survival are significantly lower in lung adenocarcinoma with STAS. Even in stage I lung adenocarcinoma, a positive STAS has been associated with a poor prognosis (2, 3, 11–13). However, STAS assessment requires postoperative pathology to make a diagnosis, and a preoperative assessment of STAS based on lung CT imaging data would be very helpful in formulating a rational treatment plan and predicting the patient’s prognosis.

Several previous studies (11, 14, 15) have shown that tumor size is significantly associated with STAS. De Margerie-Mellon et al. (14) showed that the total mean diameter and long-axis diameter of STAS-positive nodules were significantly larger than those of STAS-negative nodules (p = 0.024), and the mean and maximal diameters of the solid components of STAS-positive nodules were significantly larger than those of STAS-negative nodules (p = 0.001 and 0.003) when the solid component was ≥10 mm in long-axis diameter, and the number of STAS-positive nodules was significantly higher than that of STAS-negative nodules (p < 0.001). Toyokawa et al. (11) showed that a radiological tumor size of ≥2.0 cm was significantly associated with the presence of STAS (p = 0.007). However, Kim (16) and Liu Zhan (17) have suggested that the percentage of solid components is better in predicting STAS than the maximal diameter of solid components. In the univariate analysis in this study, the differences in CTR and the maximal diameter of the solid component between the two groups were statistically significant (p < 0.05), while the multivariate analysis showed that only the maximal diameter of the solid component was an independent risk factor of STAS. This may be related to the different selection of study subjects. Kim et al. (16) have studied lung adenocarcinoma of all stages, including carcinoma *in situ* and minimally invasive adenocarcinoma, while the subjects in this study all had invasive lung adenocarcinoma. Moreover, in the results of this study, the AUC of using the maximal diameter of the solid component to predict STAS was 0.757 (95% CI, 0.714–0.799; p < 0.001), and the sensitivity of the cutoff value of 1.18 cm was 73.9%, and the specificity was 69.1%. It is better than the prediction performance of the CTR in the study by Liu Zhan et al. (17) (AUC = 0.71) and is close to the prediction performance of the CTR in the study by Kim (16) (AUC = 0.77), suggesting that the design and results of this study have good clinical predictive value and application prospect.

Among 503 cases of invasive lung adenocarcinoma collected in this study, 241 cases (47.9%) were STAS positive, and the incidence rate was at the median level of many previous studies (14.8%–60.5%) (3, 8, 13, 18), indicating that the incidence of STAS in invasive lung adenocarcinoma is relatively high and suggesting that careful observation should be made during pathological diagnosis to guide clinicians in preparing treatment plans. Among the CT signs, this study showed that the differences in lesion density, the maximal diameter of the tumor, maximal diameter of solid component, CTR, and common malignant signs in invasive lung adenocarcinoma—such as lobulation, spiculation, cavitation, air bronchogram, pleural indentation, and vascular convergence signs—were statistically significant (p < 0.05) between the STAS-positive and STAS-negative groups, which is consistent with the results of De Margerie-Mellon (14) and Chen D et al. (16, 19). In the multivariate regression analysis, it was found that the maximum diameter of the solid component, spiculation, and cavitation were independent risk factors for STAS, providing an important reference for exploring the relationship between CT signs and the degree of invasion in lung adenocarcinoma.

The structure and density of lung cancer can reflect its pathological stage, and the more solid components shown on CT may usually indicate a more pronounced pathological infiltration. In this study, it was found that invasive lung adenocarcinoma with more solid nodules or more solid components on CT had a higher STAS-positive rate, which also verified the conclusion of Shiono et al. (3). Although Kim (16) suggested that STAS does not exist in pure ground-glass nodule (pGGN)-like lung adenocarcinoma, about 17.27% (19/110) of cases were pGGN in this study. The existence of STAS in pGGN was also found and confirmed by Jiang (20) and Zhang Zhenrong (21), which may explain the reasons for the poor prognosis of pGGN-like lung adenocarcinoma.

In terms of other relevant clinical and pathological indicators, several studies (6, 8, 11) have shown that STAS is associated with features reflecting aggressiveness, e.g., STAS-positive individuals are usually at an advanced stage of lung adenocarcinoma and are prone to lymph node metastasis and vascular invasion. Our study also found that STAS was more common in papillary, micropapillary, and solid lung adenocarcinomas, and the positive rate of STAS was also higher in T2, N2, and IB+ lung adenocarcinomas (p = 0.024, p < 0.001, p < 0.001). There were also significant differences between the STAS-positive and STAS-negative groups in terms of lymph node metastasis, pleural invasion, and neurovascular invasion (all p < 0.01).

In summary, STAS, as a newly named and pathologically diagnosed aggressive pattern of lung adenocarcinoma, not only influences the pathological diagnosis in terms of invasion but also has a major impact on the choice of surgery and other treatment methods for lung adenocarcinoma patients. The results of this study show that the largest diameter of the solid component in lung adenocarcinoma lesions on CT and CRT are still important signs for predicting STAS, and common signs of other malignancies also have some predictive value. However, this is a retrospective study, possibly presenting selection bias, and there is a lack of longitudinal follow-up observation. It is necessary for these results to be further explored and verified in future prospective and multicenter studies.

## Data availability statement

The raw data supporting the conclusions of this article will be made available by the authors, without undue reservation.

## Ethics statement

The Research Ethics Committee of Zhongshan Hospital Affiliated to Dalian University (Dalian, China) approved this study (project approval number 2021029) and waived informed consent for this retrospective study.

## Author contributions

LQ, YS, and JW conceived the study. LQ and YS collected the data. LQ analyzed the data. LQ wrote the manuscript. JW provided study supervision. BH and RZ conducted the histological assessments. All authors contributed to the article and approved the submitted version.

## Acknowledgments

The authors thank all the staff from the Departments of Thoracic surgery, Radiology and Pathology, Affiliated Zhongshan and Xinhua Hospital of Dalian University, for their help in collecting the clinical data.

## Conflict of interest

The authors declare that the research was conducted in the absence of any commercial or financial relationships that could be construed as a potential conflict of interest.

## Publisher’s note

All claims expressed in this article are solely those of the authors and do not necessarily represent those of their affiliated organizations, or those of the publisher, the editors and the reviewers. Any product that may be evaluated in this article, or claim that may be made by its manufacturer, is not guaranteed or endorsed by the publisher.
